# Deep learning insights into β-lactamase dynamics and resistance evolution

**DOI:** 10.1042/BCJ20260196

**Published:** 2026-05-08

**Authors:** Jing Gu, Lin Gao, Shuang Chen, Manming Xu, Jassi Goyal, Rida Haider, Fedaa Attana, Othman R.A. Alzahrani, Robert A. Bonomo, Shozeb Haider

**Affiliations:** 1 Department of Pharmaceutical and Biological Chemistry, School of Pharmacy, University College London, London WC1N 1AX, U.K.; 2 Department of Biophysics, University of Delhi, New Delhi 110021, India; 3 Institute of Structure and Molecular Biology, School of Natural Sciences, Birkbeck University of London, London WC1E 7HX, U.K.; 4 Prince Fahd Bin Sultan Chair for Biomedical Research, University of Tabuk, Tabuk 71491, Saudi Arabia; 5 Department of Biology, Faculty of Science, University of Tabuk, Tabuk 71491, Saudi Arabia; 6 Department of Molecular Biology and Microbiology, Case Western Reserve University School of Medicine, Cleveland, OH, U.S.A.; 7 Research Service, Louis Stokes Cleveland Department of Veterans Affairs Medical Centre, Cleveland, OH, U.S.A.; 8 Department of Biochemistry, Case Western Reserve University School of Medicine, Cleveland, OH, U.S.A.; 9 Department of Medicine, Case Western Reserve University School of Medicine, Cleveland, OH, U.S.A.; 10 Department of Pharmacology, Case Western Reserve University School of Medicine, Cleveland, OH, U.S.A.; 11 Department of Proteomics and Bioinformatics, Case Western Reserve University School of Medicine, Cleveland, OH, U.S.A.; 12 CWRU-Cleveland VAMC Centre for Antimicrobial Resistance and Epidemiology (Case VA CARES) Cleveland, OH, U.S.A.

**Keywords:** Βeta Lactamase, Deep Learning, Molecular Dynamics simulations

## Abstract

The rapid global expansion of β-lactamase-mediated antimicrobial resistance demands mechanistic approaches capable of resolving the dynamics of enzyme adaptation. Although β-lactamase evolution often involves subtle rearrangements rather than large structural shifts, traditional structural and simulation analyses struggle to capture the conformational heterogeneity that underlies shifts in substrate specificity and inhibitor susceptibility. Here, we review recent advances in applying deep learning to probe the conformational dynamics of β-lactamases across classes A–D. We highlight how convolutional variational autoencoders (CVAEs) reconstruct nonlinear conformational manifolds from molecular dynamics simulations, exposing metastable states, cryptic pockets, and catalytic intermediates. DiffNets integrate supervised objectives to identify structural determinants of biochemical phenotypes, while BindSiteS-CNN and geometric deep learning methods provide high-resolution insight into active-site remodelling and local pocket plasticity. Additionally, graph neural networks trained on dynamics-informed descriptors capture long-range allosteric couplings and accurately predict mutational fitness and epistasis. The deep learning-enabled analysis of protein dynamics offers a unified and predictive framework for understanding β-lactamase adaptation.

## Introduction

The global rise of antimicrobial resistance (AMR) represents one of the most urgent healthcare crises [1]. A central driver of this phenomenon is the widespread proliferation of β-lactamase enzymes, which inactivate β-lactam antibiotics like penicillins, cephalosporins, and carbapenems, by hydrolysing their β-lactam ring [2]. Since β-lactam antibiotics remain among the most widely prescribed, trusted and clinically valuable antimicrobial agents, the diversification of β-lactamases poses a direct and significant threat to modern medicine [2,3].

**Figure 1:**
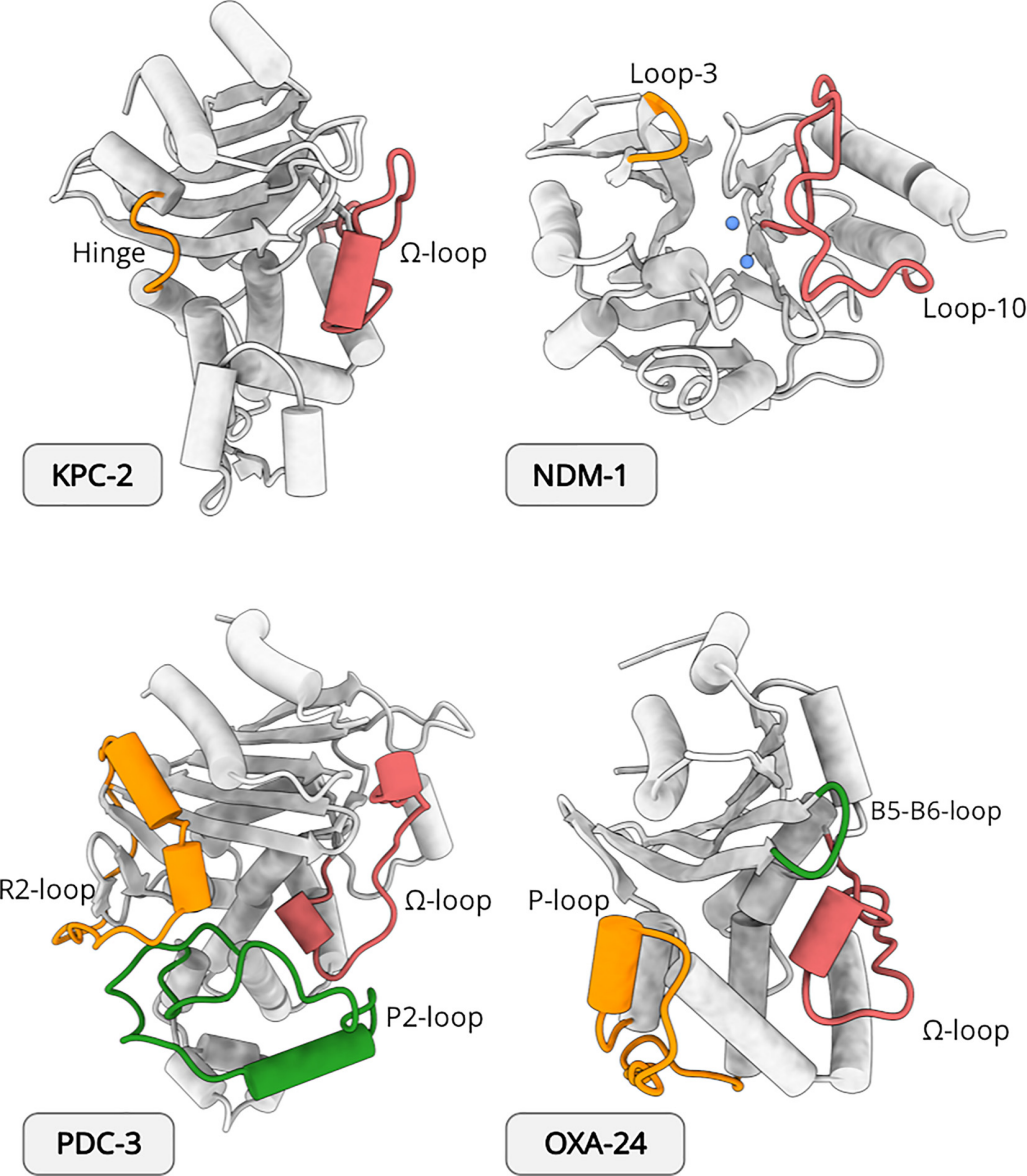
Four classes of β-lactamases Structural comparison of representative β-lactamases highlighting active-site loop regions. Cartoon representations of four clinically relevant β-lactamases are shown: KPC-2 (class A, PDB id 2OV5), NDM-1 (class B, PDB id 3SPU), PDC-3 (class C, PDB id 8SDL), and OXA-24 (class D, PDB id 3ZNT). Protein backbones are displayed in light grey, with key structural elements that shape the active site highlighted in colour. In KPC-2, the Ω-loop (pink) and hinge region (orange) are indicated. In NDM-1, Loop 3 (orange) and Loop 10 (pink) are shown; blue spheres denote catalytic Zn^2+^ ions. In PDC-3, the R2-loop (orange), P2-loop (green), and Ω-loop (pink) are highlighted. In OXA-24, the P-loop (orange), B5-B6 loop (green) and Ω-loop (pink) are indicated. These loop regions contribute to substrate recognition and active-site architecture, illustrating structural diversity among β-lactamase classes.

By Ambler classification, classes A, C and D are serine β-lactamases (SBLs), and class B are zinc-dependent metallo-β-lactamases (MBLs) [4]. Despite differences in catalytic chemistry, all classes share a defining characteristic involving remarkable evolutionary adaptability, where single amino acid substitutions, insertions, and deletions reconfigure conformational landscapes across the enzyme scaffold (Figure 1) [5]. Interestingly, functional diversification in β-lactamases is rarely driven by dramatic structural rearrangements [6]. Instead, subtle shifts in loop positioning, helix packing, hydrogen-bond networks, and hydrophobic cores influence dynamic communication pathways that modulate substrate specificity and compromise inhibitor susceptibility [7–11]. These changes often involve minor structural variations that are difficult to detect by direct comparison of static crystal structures. Consequently, a mechanistic understanding of resistance evolution requires approaches capable of resolving conformational heterogeneity at atomic resolution and across extensive timescales.

Molecular dynamics (MD) simulations have emerged as a powerful method to characterise enzyme flexibility and rare transitions [12–17]. Recent advances in computing power have increased trajectory length and complexity to such an extent that post-simulation analysis has become a bottleneck [18–22]. Classical dimensionality reduction techniques such as principal component analysis (PCA) [23–25] or time-lagged independent component analysis (tICA) [26–28] provide valuable insights but are inherently limited by linear assumptions or predefined feature choices. To overcome these challenges, machine learning has transformed the analysis of biomolecular simulation data [29–31]. Deep learning-based techniques can extract nonlinear, high-dimensional patterns from MD ensembles [32,33], revealing hidden conformational states [9,34], allosteric sites and networks [35–37], and subtle dynamic signatures associated with functional divergence [10,38].

It is important to note that deep learning has also begun to play a role in the discovery of β-lactamase inhibitors. Several recent studies have employed machine learning and deep learning models to predict inhibitor activity, guide virtual screening, and identify novel scaffolds targeting enzymes such as AmpC and MBLs [39–43]. However, most of these efforts focus on activity prediction and hit prioritisation rather than fully generative, *de novo* inhibitor design, and remain complementary to experimental medicinal chemistry efforts. While such applications are promising and represent an emerging frontier in antimicrobial drug discovery, they are not the primary focus of the present review.

This review instead focuses on dynamics-driven key deep learning pipelines that have proven especially informative in β-lactamase research, including DiffNets, convolutional variational autoencoders (CVAEs), BindSiteS-CNN and graph neural networks (GNN). We then discuss how these methods have been applied to four classes of β-lactamases, highlighting case studies that link conformational dynamics to resistance phenotypes and mechanistic adaptation, with implications for inhibitor development.

## Deep learning methods in β-lactamase research

### Convolutional variational autoencoders

Dimensionality reduction is central to the interpretation of MD simulations [44]. Extracting mechanistically meaningful patterns from MD simulation datasets requires dimensionality reduction methods that preserve nonlinear structural relationships while maintaining sampling heterogeneity. Autoencoders represent one of the earliest neural network architectures for nonlinear dimensionality reduction [45]. A conventional autoencoder consists of an encoder that maps an input structure to a latent vector and a decoder that reconstructs the original input [45]. Training minimises reconstruction loss, typically measured as mean squared error or binary cross-entropy [46]. However, standard autoencoders lack explicit regularisation of the latent space, often producing irregular embeddings that generalise poorly and may overfit individual conformations [47].

Variational autoencoders extend this approach by imposing a probabilistic constraint on the latent variables. Instead of mapping each input to a fixed point, the encoder outputs the parameters of a predefined distribution that is a multivariate Gaussian [48]. The training objective combines reconstruction loss with a Kullback–Leibler (KL) divergence penalty, which regularises the latent distribution toward a prior [49]. This constraint encourages the model to learn a continuous, smooth latent manifold that reflects general structural principles rather than memorising individual structures.

**Figure 2:**
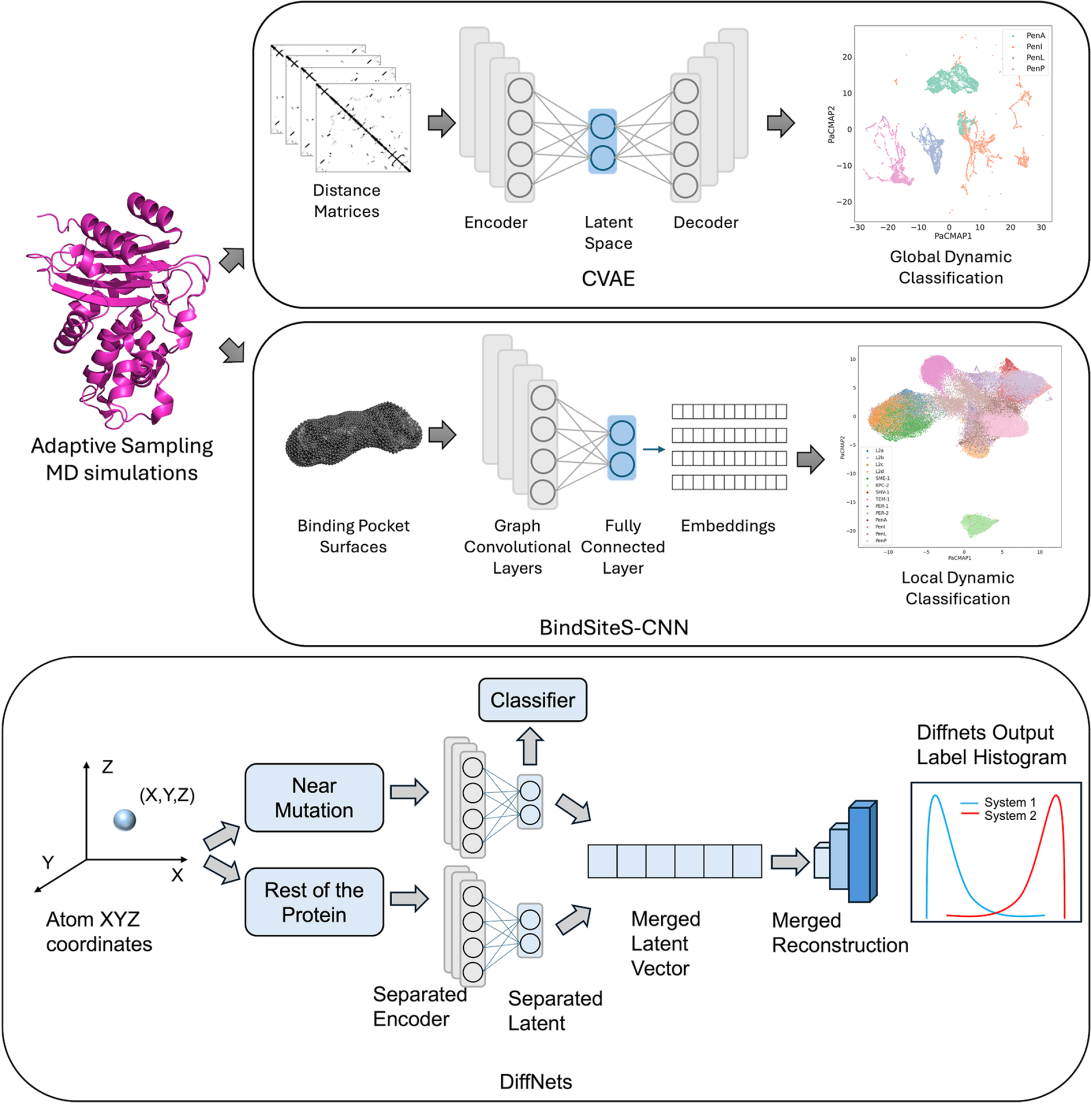
Overview of deep learning pipelines for analysing protein dynamics from adaptive sampling MD simulations Distance matrices from MD trajectories are input to a CVAE to learn latent representations and classify global conformational dynamics, while binding pocket surfaces are processed with graph convolutional layers in the BindSiteS-CNN model to generate embeddings that detect and classify local structural changes in binding pockets. DiffNets add functional signature to resolve learned latent coordinates.

CVAEs adapt this architecture for structured inputs such as residue-residue contact maps or Cα–Cα distance matrices derived from MD simulations [34]. The convolutional layers progressively extract spatially correlated features from the distance matrices. These features capture both local secondary structure interactions and long-range contact networks like recurring structural motifs such as loop closures, helix-sheet packing, or domain motions. The encoder outputs parameters of a latent Gaussian distribution, and sampling from this distribution defines a low-dimensional embedding of each MD simulated frame. The variational constraint regularises the latent space, ensuring that similar conformations occupy contiguous regions of the manifold. When MD trajectories are projected into this space, metastable conformational states often form compact clusters, while transition pathways appear as continuous trajectories between basins. The resulting latent embeddings can be clustered, visualised, and interpreted in terms of metastable conformational states. The decoder then reconstructs the original distance matrix, and training minimises a hybrid loss combining reconstruction error and KL divergence. CVAEs are particularly well suited for MD trajectory analysis for several reasons: (a) distance matrices provide rotation- and translation-invariant structural representations, (b) convolutional filters capture multiscale structural motifs without manual feature selection, and (c) the variational bottleneck produces a continuous and physically interpretable conformational manifold [34]. When integrated with adaptive sampling, CVAEs facilitate reconstruction of free-energy landscapes and identification of rare intermediate states that may correspond to catalytically competent or inhibitor-sensitive conformations (Figure 2) [50–52].

### DiffNets for function-orientated MD embedding

Standard unsupervised autoencoders compress high-dimensional structural inputs into a low-dimensional latent space by minimising reconstruction error [45]. While effective at capturing dominant geometric motions, such models often emphasise large-scale structural differences at the expense of subtle yet functionally critical rearrangements, i.e., they do not explicitly distinguish which structural variations are functionally relevant. In MD simulations of enzyme variants, ensembles frequently overlap substantially in conformational space, even when biochemical properties differ. As a result, purely unsupervised embeddings may fail to highlight subtle structural determinants of phenotype.

DiffNets address this challenge by combining dimensionality reduction with a supervised classification objective [53]. In addition to reconstructing input structures, the network is trained to predict variant-level labels (e.g., stable versus unstable, susceptible versus resistant). This additional constraint reshapes the latent space so that structural features correlated with functional differences become separable. A key advance of DiffNets is the incorporation of expectation–maximisation (EM) scheme that iteratively refines frame-level label probabilities during training [54]. Rather than assuming all frames from a variant uniformly reflect its phenotype, the EM procedure estimates which conformations are most predictive of the observed biochemical property. This approach reshapes the latent space so that conformations associated with a functional signature, such as helix compaction, loop stabilisation, or altered inter-domain packing, become separable along learned latent coordinates.

### BindSiteS-CNN and geometric deep learning

**Figure 3:**
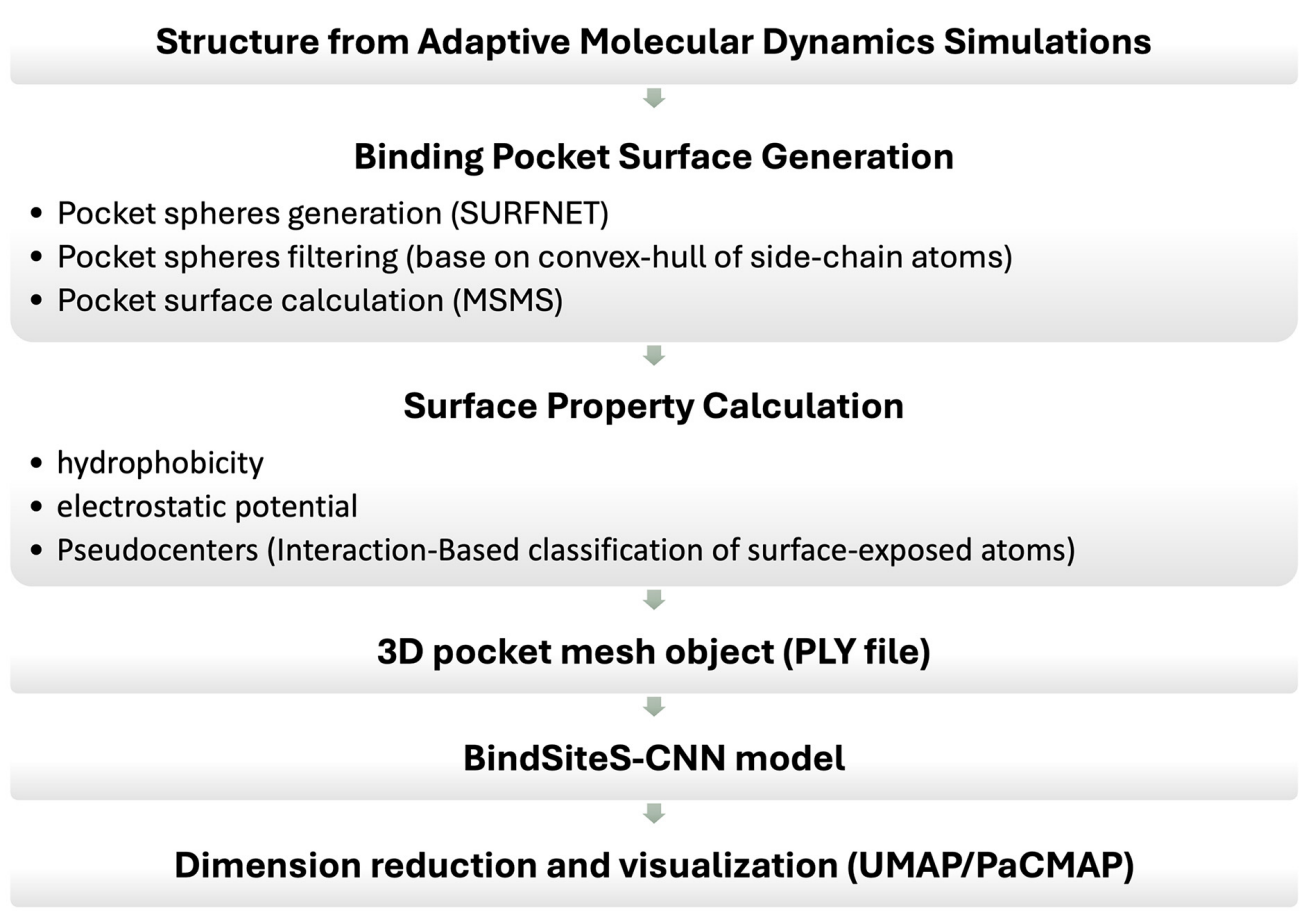
Workflow for local binding pocket analysis employing BindSiteS-CNN model BindSiteS-CNN model produces embeddings that are visualised using dimensionality reduction methods (UMAP/PaCMAP) to identify distinct pocket conformational states.

Although CVAEs and DiffNets capture global backbone dynamics from MD simulations, enzymatic specificity and inhibitor compatibility often depend on localised rearrangements within the catalytic pocket [54,55]. Thermal fluctuations of side chains, subtle reshaping of cavity volume, and reorganisation of electrostatic networks may not be fully resolved through backbone-centric embeddings. BindSiteS-CNN and related geometric deep learning approaches address this limitation by analysing MD-derived binding-site conformations directly [56–59]. From each simulation frame, the active-site region is extracted and projected onto a spherical surface centred on the pocket. This surface is annotated with physicochemical descriptors such as hydrophobicity, electrostatic potential, and hydrogen-bond donor/acceptor distributions, computed from atomic coordinates. Spherical convolutional neural networks then process these rotation-equivariant representations [60], enabling consistent comparison of pocket geometry across MD frames without requiring structural alignment. Clustering of these embeddings reveals state-specific pocket conformations that may correspond to open, constricted, or inhibitor-compatible geometries (Figure 3).

When integrated with CVAE or DiffNet embeddings of the full protein, BindSiteS-CNN provides a complementary, local perspective on MD trajectories. The combined approaches link global conformational shifts to localised catalytic-site remodelling, enabling multi-scale interpretation of how sequence variation or ligand binding alters both overall dynamics and active-site architecture.

### Graph neural networks

A GNN is a deep learning architecture in which entities are represented as nodes and their pairwise relationships as edges, enabling the propagation of information across the network through successive message-passing layers [61]. This maps naturally onto protein topology, where amino acid residues serve as nodes and their spatial or dynamic relationships define the edges. Each residue’s learned embedding is progressively refined through updates from its neighbouring residues and the features of the connecting edges. After multiple propagation steps, the final representation implicitly captures information integrated from across the complete protein structure.

The elastic network model (ENM) provides a natural foundation for constructing such graphs. In ENM, residues (often Cα atoms) are treated as nodes connected by elastic springs to approximate intrinsic fluctuations and large-scale collective motions [62,63]. This simplified harmonic implementation enables efficient normal-mode analysis to estimate protein dynamics without the computational cost of full all-atom MD simulations [64]. A physics-based descriptor, asymmetric dynamic coupling index (DCI_asym_), that integrates perturbation response scanning and linear response theory with ENM, quantifies how perturbations in one residue influence the flexibility of another across long distances in the structure [65–67]. The collective motions make the resulting residue–residue couplings inherently suitable as structured numerical data for downstream deep learning applications. The ENM-derived dynamic couplings can then be used to train an allosteric GNN [68]. This architecture enables the model to capture allosteric communication pathways and non-local epistatic interactions without requiring explicit training on experimental epistasis measurements.

## Applications of deep learning to β-lactamase structural dynamics

### Class A β-lactamases

Class A β-lactamases include clinically significant enzymes such as TEM-1, KPC-2, SHV-1, and SME-1. Although, they share a conserved serine-dependent catalytic mechanism, they differ widely in substrate profile and inhibitor sensitivity, often due to changes in their dynamic landscapes.

### Stable variants of TEM-1

DiffNets were used to identify subtle, functionally relevant structural signatures within MD simulations of TEM-1 β-lactamase variants [53]. TEM-1, first described in the early 1960s, remains one of the most extensively studied β-lactamases after more than six decades of investigation [69]. TEM-1 confers bacterial resistance primarily to penicillins and early-generation cephalosporins, and while mutations expand substrate profiles, they often destabilise the enzyme. The compensatory mutation M182T is frequently observed in clinical isolates and enhances stability [70]. Previous structural analyses, including crystallography, failed to reveal a clear mechanistic basis for this stabilisation [71]. However, combined simulation, NMR, and crystallographic studies demonstrated that stabilisation correlates with compaction of helix 9, characterised by strengthened intra-helical hydrogen bonds and sub-angstrom distance reductions between hydrogen-bonding partners [70–72]. Since these changes are geometrically subtle relative to surrounding loop motions, they present a stringent test for representation learning methods.

To assess whether supervised dimensionality reduction improves detection of such subtle features, the authors compared DiffNets with standard unsupervised autoencoders. A curated dataset of 178402 MD frames from wild-type and M182T simulations was constructed, evenly balanced between compact and extended helix 9 conformations. Both models used identical split encoder architectures, but DiffNets included an additional classification layer trained to distinguish compact from extended helices. Reconstruction accuracy was comparable (∼1 Å RMS error), demonstrating that both approaches effectively compressed structural data. However, their latent representations differed substantially. In unsupervised autoencoders, compact and extended states overlapped in latent space, and post hoc logistic regression yielded near-random classification performance (AUC = 0.54). In contrast, DiffNets clearly separated the two conformational classes and achieved strong classification performance (AUC = 0.91), indicating that incorporating a classification objective reorganises latent space to emphasise structurally meaningful distinctions.

The approach was then extended to a more realistic, self-supervised setting involving four variants (highly stable M182T and M182S, and less stable wild-type and M182V). Here, explicit structural feature (such as helix 9 compaction) was not provided to the model. Instead, variant-level stability labels were used, and frame-level labels were iteratively refined using EM scheme [73]. This procedure allowed overlapping conformational ensembles while constraining plausible proportions of stabilising frames. Without EM, the model effectively memorised ensemble identity, assigning extreme labels (0 or 1) to nearly all frames. With EM, predicted labels spanned a continuum, correlating smoothly with helix compaction and capturing the graded structural contributions to stability.

Importantly, the EM-trained DiffNets improved prediction of an unseen variant (M182N), supporting its capacity to learn transferable structural determinants of stability. Model interpretability was achieved by correlating DiffNets output labels with interatomic distances near the mutation site. This analysis confirmed helix 9 compaction as a dominant stabilising feature and further revealed tighter packing between helix 9 and the adjacent β-sheet that had previously been suggested experimentally but not detected computationally.

Despite its effectiveness as a comparative method for two systems, one notable limitation of the DiffNets method arises when it is applied to comparisons involving more than two protein systems. Specifically, DiffNets is fundamentally designed as a binary classification framework that categorises MD frames into two distinct labels: 0 and 1 during training. When multiple systems are analysed simultaneously, they are typically grouped into one of these two classes. As a result, the network learns structural features that distinguish the two aggregated classes rather than differences specific to individual systems within each group. If the systems within the same class exhibit heterogeneous structural characteristics, the signals associated with individual variants may become diluted or obscured, making it difficult to attribute the learned features to a particular system. Consequently, while DiffNets can effectively identify structural determinants that differentiate two functional groups, its ability to resolve fine-grained differences among multiple individual systems is limited without additional strategies such as pairwise comparisons or modified multiclass architectures. This lack of granularity not only impedes interpretability but also affects the scalability of the method. As the complexity of the network escalates and additional systems are incorporated, the binary labels become increasingly uninformative, hindering the derivation of meaningful insights from the generated embeddings. This limitation highlights the necessity for a more sophisticated labelling approach that can accommodate a wider array of categories, thereby enhancing the interpretability of the results.

### The role of hydrophobic nodes

**Figure 4:**
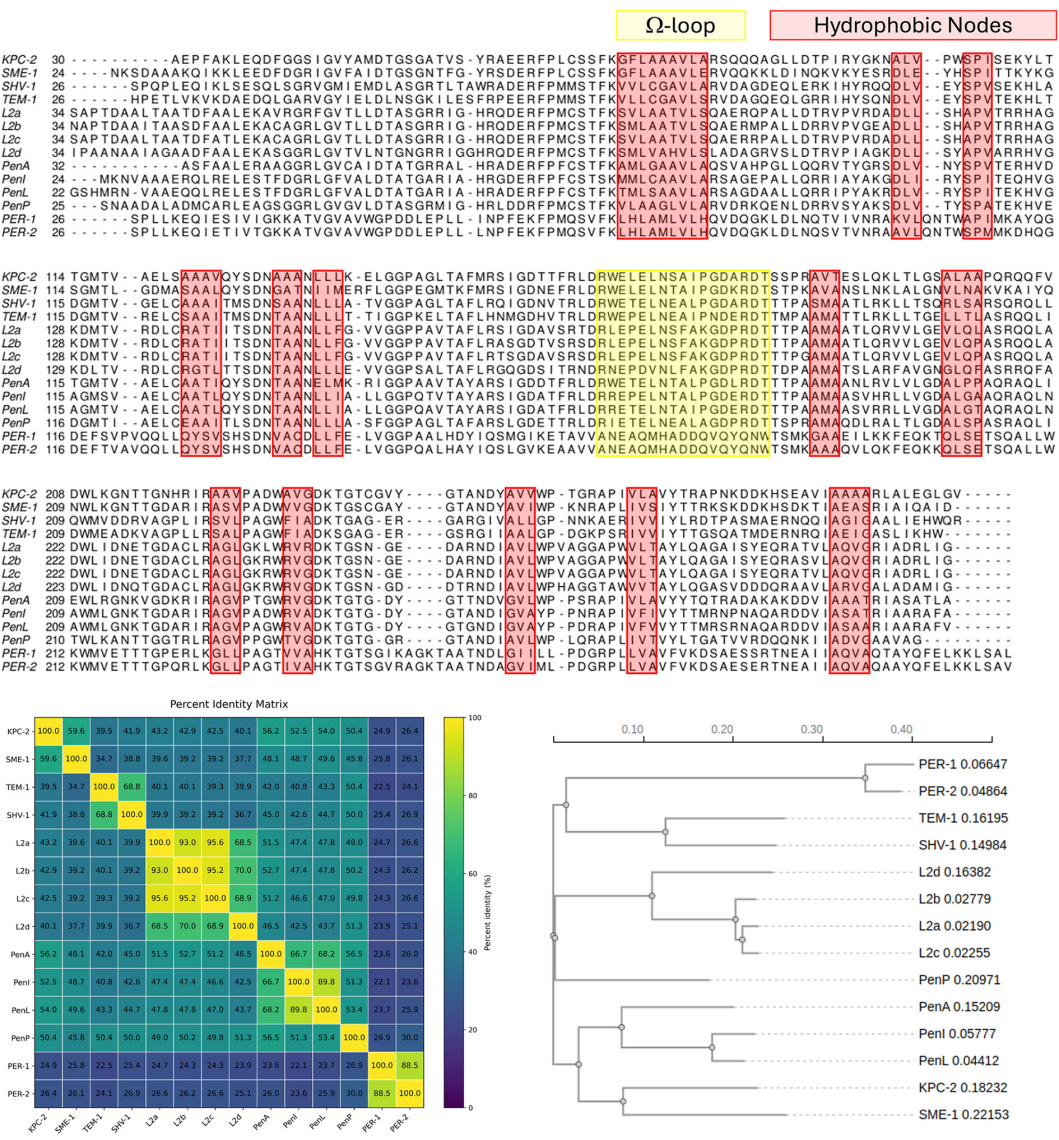
Sequence conservation and phylogenetic relationships among representative class A β-lactamases Multiple sequence alignment of selected β-lactamases highlighting the hydrophobic nodes (red) and the Ω-loop (yellow). Conserved motifs and hydrophobic residues forming the structural core are evident across enzyme families despite overall sequence divergence. Branch lengths in the phylogenetic tree represent sequence divergence, and clustering reflects known family groupings. Together, these analyses highlight conserved structural determinants alongside evolutionary diversification within β-lactamases.

The hydrophobic nodes are clusters of conserved residues forming the structural backbone and shared across class A β-lactamases (Figure 4) [10,74]. They were first reported by Galdadas et al. [74]; however, their functional role across class A family was defined by Olehnovics et al., using combined adaptive MD simulations, Markov state models and unsupervised deep learning [10]. Rather than focusing solely on static structural comparisons, the study centred on identifying hydrophobic residue clusters that act as dynamic regulators within the enzyme scaffold and elucidating how these nodes shape conformational landscapes linked to catalysis. Additional analyses reveal the coexistence of conserved structural features and evolutionary diversification within class A β-lactamases (Figure 4). Although hydrophobic nodes and Ω-loop region are largely conserved across sequences, pairwise percent identity analyses demonstrate substantial sequence divergence. This variation reflects the evolutionary relationships among homologues and supports the clustering observed in the identity matrix.

Adaptive sampling MD simulations across four representative class A β-lactamases (KPC-2, SME-1, TEM-1, and SHV-1) explored the conformational landscapes across timescales relevant to catalytic transitions [75,76]. These simulations generate high-dimensional structural ensembles reflecting both backbone and side-chain fluctuations in secondary structural elements, loops, and hydrophobic cores. An unsupervised CVAE deep learning model is then applied to the inter-residue distance data representing the hydrophobic network of each enzyme. By treating distance matrices as structured inputs, the convolutional layers capture spatial correlations in local and long-range interactions without requiring manual feature selection.

Within this latent space, conformations were embedded according to the dynamic behaviour of conserved hydrophobic nodes. Subsequent visualisation of the latent embeddings using methods such as t-distributed stochastic neighbour embedding revealed distinct clustering patterns corresponding to enzyme-specific dynamics [77]. Deep learning in the present study functioned not as a mere classifier but as a nonlinear dimensionality reduction tool that captures complex correlated motions within the hydrophobic network. By projecting high-dimensional MD-derived distance matrices into a tractable latent space, the CVAE enabled comparison of enzymes based on dynamic similarity rather than static homology. For instance, patterns in hinge region flexibility and loop repositioning correlate with differences in the functional dynamics of hydrophobic nodes, even when traditional structural analysis alone would not distinguish these features. The analysis revealed that variations in hydrophobic node flexibility and coupling correlate with functional differences among β-lactamases, highlighting these conserved hydrophobic clusters as key determinants of dynamic architecture and catalytic modulation.

### The co-evolutionary dynamics in the L2 β-lactamase family

The integrated computational workflow leveraging adaptive sampling MD simulations and deep learning models has also been used to investigate the conformational and coevolutionary dynamics of L2 β-lactamases [78]. The L2, class A β-lactamase family expressed by *Stenotrophomonas maltophilia* contributes substantially to AMR [79,80]. The deep learning components are central to the study’s analysis, enabling high-dimensional characterisation of structural ensembles derived from simulation data and the extraction of functionally relevant features without predefined structural metrics.

**Figure 5:**
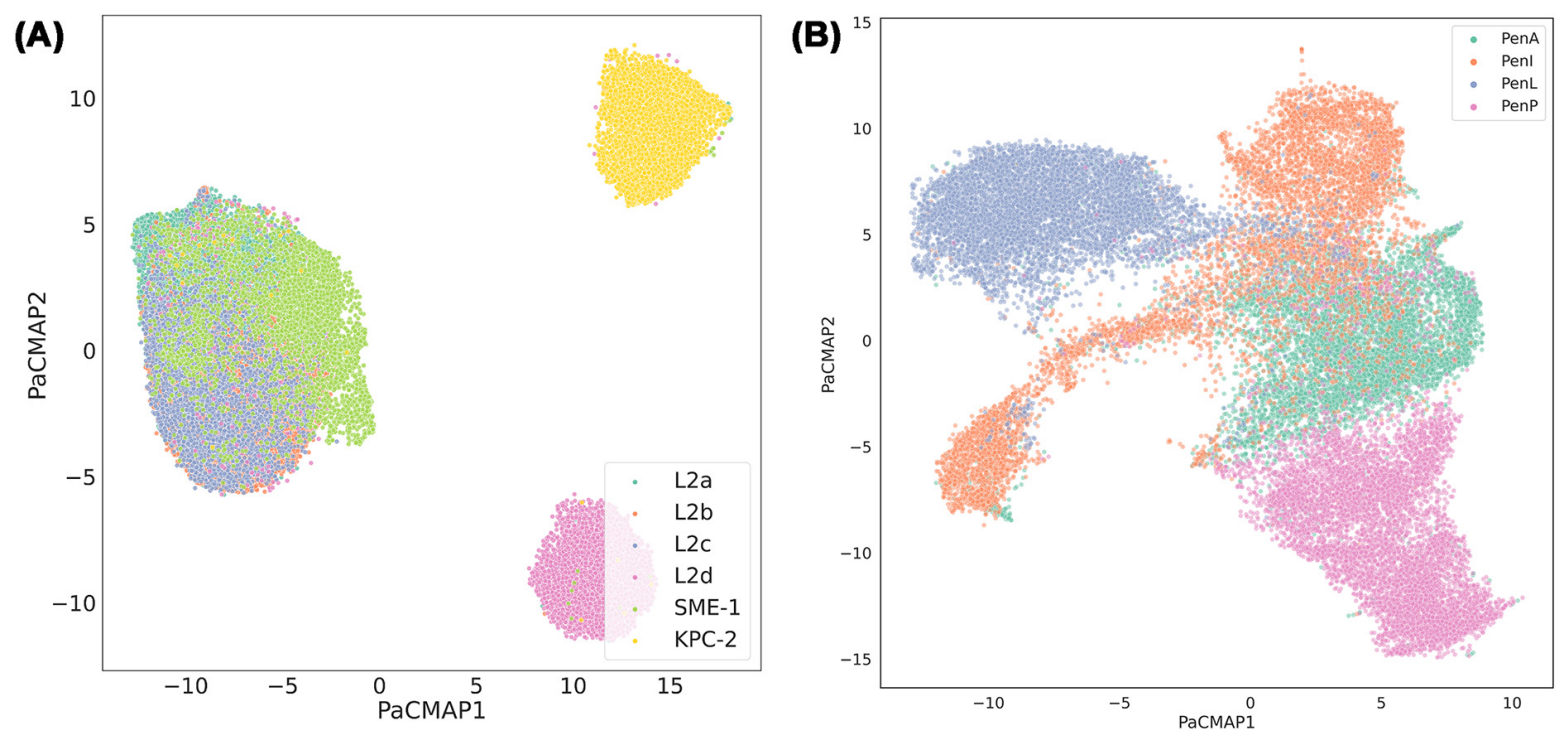
Low-dimensional embeddings of the binding pocket conformations generated by BindSiteS-CNN model and visualised using PaCMAP Each point represents a pocket structure sampled from MD simulations, revealing clusters that correspond to distinct conformational states. The separation of clusters highlights how local pocket geometry and physicochemical features differentiate functional binding modes. PaCMAP projection for (**A**) L2-family β-lactamases (L2a–L2d) together with representative class A carbapenemases (KPC-2 and SME-1) and (**B**) Pen-family β-lactamases (PenA, PenI, PenL, and PenP). Distinct clustering patterns are observed among subfamilies, structural diversification of the binding site within the Pen family.

The core deep learning methodology employs CVAE to analyse inter-residue distance matrices generated from extensive MD trajectories, capturing both local and long-range interactions [34]. In parallel, BindSiteS-CNN architecture is used to compare binding site geometries across conformational ensembles, capturing subtle variations that may underlie functional divergence or metrics of substrate specificity [58]. Together, the CVAE and BindSiteS-CNN models allow unsupervised classification of dynamic behaviour across multiple representative β-lactamases, including L2 family, SME-1, and KPC-2 (Figure 5A). The CVAE elucidates global conformational shifts and identifies clusters corresponding to distinct dynamic states, while BindSiteS-CNN identifies how local active-site geometry coevolves with global dynamics. This multiscale analysis reveals that hydrophobic nodes and specific pocket residues act as dynamic anchors, coordinating fluctuation patterns that differentiate functional phenotypes within the L2 β-lactamase family.

Interestingly, the deep learning models also enable coevolutionary mapping by correlating structural dynamics with sequence variation across the enzyme family. The latent embeddings are interpreted in conjunction with evolutionary information to highlight how conserved hydrophobic interactions and binding site motifs evolve, shaping the dynamic landscape of β-lactamase function. This integrative approach bridges the genotype–phenotype gap, offering mechanistic insight into how evolutionary pressures sculpt conformational ensembles that modulate catalytic behaviour. For validation, the learned representations derived from CVAE and BindSiteS-CNN are visualised using Pairwise Controlled Manifold Approximation (PaCMAP) dimensionality reduction to compare structural embeddings across class A enzymes, demonstrating separation of conformational clusters that correspond to distinct dynamic signatures [81]. These clusters are associated with differences in flexibility and pocket architecture that correlate with functional divergence.

### Hot spots and loop dynamics in Pen families

The evolutionary dynamics and functional differentiation of four clinically relevant Pen β-lactamases (PenA, PenI, PenL, and PenP) from *Burkholderia* species were investigated by integrating reinforcement-learning-driven enhanced sampling MD simulations with Markov state models and deep learning architectures, including CVAE and BindSiteS-CNN [38,58]. The study reported how low sequence identity among these homologous enzymes translates into distinct dynamic behaviours that underlie differences in substrate specificity and catalytic turnover, despite shared catalytic mechanisms.

Exhaustive conformational ensembles for each Pen enzyme were generated using the Adaptive Bandit MD simulation protocol [15]. Structural metrics like root-mean-square fluctuation and dynamic cross-correlation analyses highlight that, although Pen β-lactamases have conserved overall folds, they exhibit pronounced variability in loop dynamics and core flexibility. These differences occur particularly in regions such as the Ω-loop and β9–α12 loop, which are known to influence substrate accessibility and active-site geometry in class A β-lactamases.

To systematically extract mechanistic insight from the high-dimensional MD trajectories, the study employs CVAE. When projected into two dimensions via PaCMAP [82], these latent embeddings reveal distinct dynamic basins that correspond to alternative conformational substates within each Pen β-lactamase. In particular, CVAE latent projections uncover differences in the conformational sampling of key structural motifs, such as the central section of the Ω-loop or the β9–α12 loop, that distinguish the dynamic signatures of PenA, PenI, PenL, and PenP. These latent representations are subsequently related to functionally relevant dynamics, including loop opening/closing motions that modulate catalytic site exposure. Complementing the CVAE analyses, the study utilises BindSiteS-CNN to investigate local active-site environments. Subsequent dimensionality reduction of the BindSiteS-CNN embeddings shows clustering of pocket conformations that reflect local geometric differences among Pen enzymes, such as variations in pocket volume and loop orientations (Figure 5B). The BindSiteS-CNN output thus provides a fine-grained view of how local active-site architecture coevolves with global conformational dynamics.

Integrative analysis of the CVAE and BindSiteS-CNN outputs reveals that dynamic differences among Pen β-lactamases are not merely attributable to static structural variation but rather arise from coordinated alterations in both global motions and local active-site microstates. The models identify critical sequence positions and structural hot spots that correlate with differential flexibility, which may influence observed substrate profiles and catalytic efficiency differences. Such data-driven insights extend beyond traditional structural comparison, demonstrating that deep learning-enabled analysis of MD trajectories can effectively map evolutionary dynamics to functional phenotype in β-lactamase families.

### Deep mutational scanning using Graph Neural Networks

Dynamics-informed GNNs have demonstrated substantial improvements in predicting the functional consequences of mutations in TEM β-lactamase family [68]. The conformational ensembles were generated, and the ENM-derived descriptor, DCI_asym_, was used to quantify long-range dynamical interactions between residue pairs [65]. Although the approach is a physics-based harmonic approximation, the key significance lies not in the methodological details but in the predictive outcomes. These ENM-derived couplings summarise residue-residue communication in a manner that captures essential aspects of protein flexibility, providing a rich, structured dataset that can be leveraged in deep mutational scanning applications to β-lactamases.

The most noteworthy result is that the allosteric GNN was trained solely on ENM-derived interaction patterns and consistently outperformed existing predictors of mutational fitness and epistasis across multiple experimental datasets. Importantly, this improvement holds even though the model was not trained on experimental epistasis measurements, highlighting that ENM-based dynamic features encode biologically meaningful information about cooperative mutational effects. This approach shows exceptional sensitivity to epistatic interactions, particularly those occurring between residues that are spatially distant from the catalytic pocket. The predictions are validated on 37 novel, computationally designed TEM-1 β-lactamase variants of previously unknown function. The model’s predictions show strong agreement with experimental measurements, demonstrating that ENM-derived dynamical features, despite being coarse-grained, capture determinants of β-lactamase activity. These long-range effects are central to the behaviour of many β-lactamases, where distal mutations frequently reshape active-site properties or alter substrate specificity through long-range communication networks [7]. The model’s success therefore indicates that dynamics-derived representations can reveal precisely the kind of hidden constraints and evolutionary couplings that deep mutational scanning seeks to quantify [83].

## Class B β-lactamases

### The dimer-of-dimer dynamics in L1 MBL

The L1 MBL belongs to the Ambler class B3 subclass, confers resistance to a broad range of β-lactam antibiotics and is resistant to clinically available β-lactamase inhibitors except monobactam [84]. L1 MBL assembles as a homotetramer, with each monomer containing a dinuclear Zn^2+^ active site bridged by a hydroxide/water molecule that functions as the nucleophile in β-lactam hydrolysis. The catalytic centre is flanked by two extended surface loops, α3–β7 and β12–α5, which contributes to dynamic gating that regulates substrate access and product release. These loops make multiple interactions and undergo coordinated rearrangements to reshape the catalytic cavity.

In the closed conformation, the β12–α5 loop collapses over the catalytic cavity and forms a stabilising interaction network with α3–β7. A D150c-R236 salt bridge and π–π stacking between H151 and Y227 anchor the loops in a compact arrangement. P225 adopts an inward orientation, positioning its Cγ atom approximately 6.5 Å from the zinc centroid, contributing to active-site occlusion. Closure is further stabilised by an interaction between Q310 on the α6 helix and the backbone carbonyl of A224, effectively tethering the gating loops and producing a tightly sealed catalytic pocket [85].

To quantify these conformational transitions, adaptive MD simulations were integrated with Markov state models [76]. Backbone torsions and Cα-RMSD descriptors for the two loops resolved seven metastable states spanning a rugged free-energy landscape. These states partition into three dominant ensembles, namely closed, intermediate, and open. The closed state represents the lowest-energy basin and is stabilised by the D150c-R236 salt bridge, H151-Y227 stacking, and inward P225 orientation. Disruption of these interactions leads to intermediate and fully open conformations characterised by loop displacement, α6-helix tilting, and expansion of the catalytic cavity to permit ligand access. Flux analysis revealed a dominant low-barrier transition pathway connecting open, intermediate, and closed states, accounting for ∼90% of equilibrium population flow and providing a kinetic description for loop gating.

To further investigate loop dynamics, CVAE was applied encompassing both gating loops in individual subunits. The CVAE learned a nonlinear latent representation of correlated loop motions without predefined reaction coordinates, enabling unsupervised clustering of conformations. Latent space organisation distinctly separated open, intermediate, and closed states while revealing dimer-of-dimer motions within the tetramer [85]. Importantly, the CVAE latent variables capture correlated loop motions rather than simple geometric displacement, highlighting that gating involves cooperative interactions between α3–β7 and β12–α5 loops. Correlation analysis of latent embeddings identified residues D150c, H151, P225, Y227, and R236 as key stabilising interactions in closed conformations. Projection of MSM-derived states onto the CVAE latent space established direct correlation between physically interpretable gating conformations and deep learning clusters. The multiscale description of dynamics using deep learning illustrated how conformational plasticity governs catalytic competence in L1 MBL.

## Class C β-lactamases

### Cryptic pockets in PDC-3 β-lactamase

Class C β-lactamases (AmpC enzymes) present a central challenge for mechanistic enzymology and inhibitor design because catalytically decisive conformational states are frequently transient, cryptic, and under-represented in crystal structures or in ensembles generated using classical MD simulations [86]. In the *Pseudomonas*-derived cephalosporinase PDC-3, the Ω-loop constitutes both a major clinical mutational hotspot and a key determinant of active-site plasticity [87]. Ω-loop dynamics were investigated by analysing PDC-3 and nine clinically relevant variants (V211A/G, G214A/R, E219A/G/K, Y221A/H) using an integrated enhanced-sampling and unsupervised deep learning workflow designed to reconstruct the conformational landscape and relate it to biochemical function [50].

**Figure 6:**
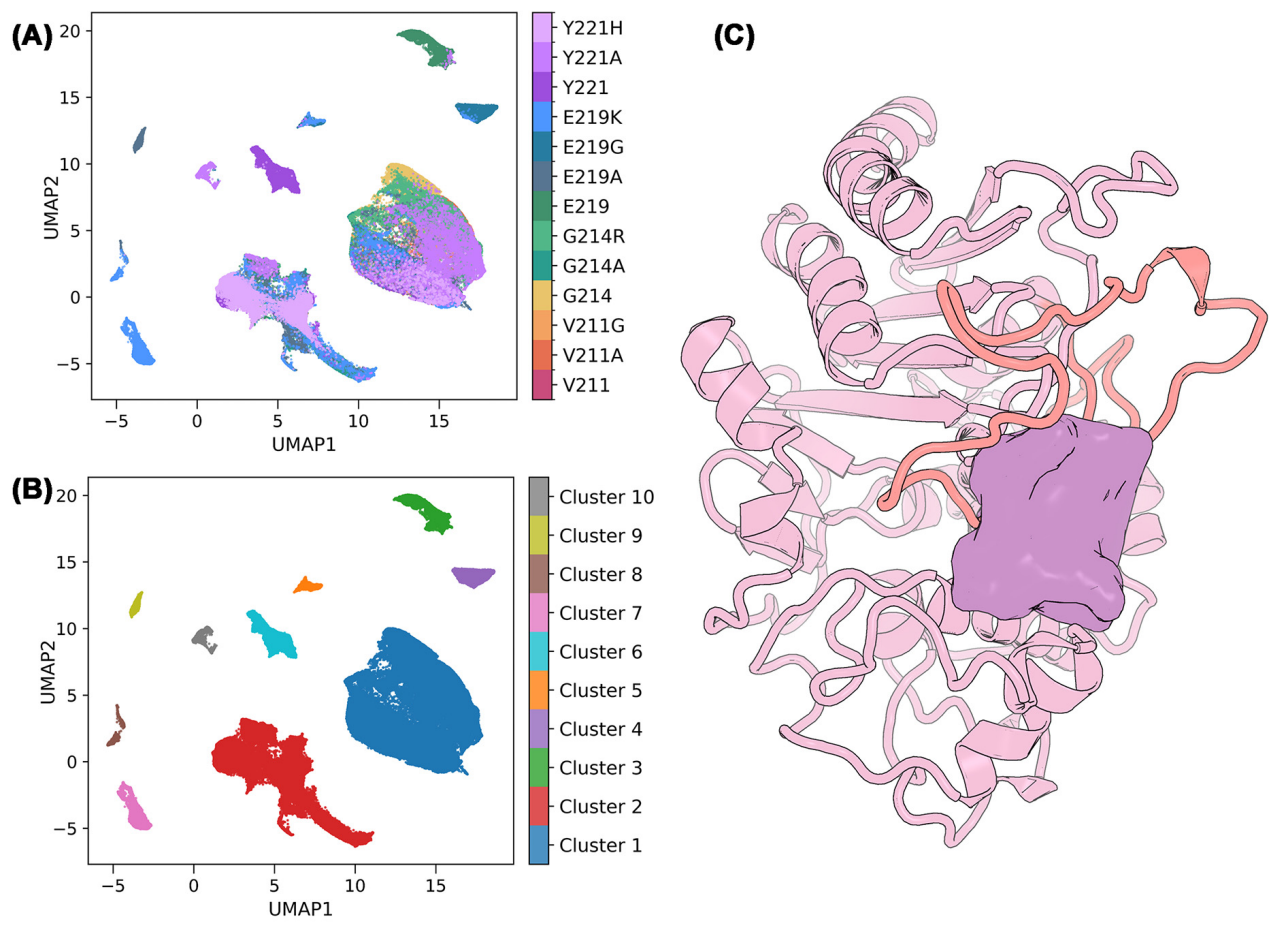
CVAE–UMAP clustering reveals distinct Ω-loop conformational states in PDC-3 β-lactamase (**A**) 2D UMAP projection of CVAE latent embeddings for all low-energy frames, coloured by variant identity. Each point corresponds to a single structural conformation sampled from residue 211, 214, 219, and 221 torsion biases (including wild-type and mutants). (**B**) The same UMAP projection coloured by HDBSCAN cluster assignment (clusters 1–10). (**C**) A representative constricted-state conformation of PDC-3 (cluster 9). The Ω-loop (salmon) shifts inward to occlude the catalytic site, revealing a previously unobserved cryptic pocket on the Ω-loop’s exterior, depicted as a mauve surface.

Sampling limitations were addressed using well-tempered metadynamics [88], biasing backbone φ/ψ dihedrals at flexible and mutation-prone Ω-loop positions (211, 214, 219, 221). In each system, biasing targeted either the wild-type residue or its substitution. Converged free-energy surfaces were used to extract low-energy conformations for downstream analysis. This separation of sampling and learning ensured that the neural network was trained on ensembles enriched in rare but mechanistically relevant transitions, rather than attempting to infer poorly sampled states (Figure 6A).

A key design choice for applying deep learning to protein ensembles is how to featurise structures in a way that is information-rich yet robust to trivial symmetries. Structural frames were encoded as fixed-size Cα–Cα distance matrices spanning conserved catalytic motifs, the Ω-loop, helix 10, and additional residues shaping the active-site cleft. CVAE was trained as an unsupervised generative model. The metastable conformations formed compact neighbourhoods in latent space, and transitions followed continuous trajectories rather than stochastic scatter. Each conformation is therefore represented by an eight-dimensional latent vector that effectively functions as a learned, nonlinear collective variable capturing different Ω-loop motion.

Latent embeddings were visualised using UMAP [81] and clustered via HDBSCAN (Figure 6B) [89], yielding three principal Ω-loop ensembles: (i) a crystal-like state resembling the experimental structure; (ii) an expansive state in which outward loop displacement widens the active-site cleft; and (iii) a constricted state characterised by inward motion that narrows or partially occludes catalytic access. These clusters were mechanistically interpretable. Distances corresponding to hallmark interactions, including the K67-G220 hydrogen bond and the Y150-A292 contact, displayed sharply separated distributions across ensembles, indicating that the CVAE captured functionally meaningful rearrangements rather than trivial variance.

Beyond state classification, the model revealed recurrent formation of a D217-K67 salt bridge in expansive substates, reminiscent of the general base E166 in class A β-lactamases [5]. QM/MM free-energy calculations supported a feasible water-mediated proton-transfer relay involving K67, S64, and D217, suggesting a conformation-dependent backup general base mechanism. Additionally, constricted states exposed a previously unrecognised cryptic pocket on the exterior face of the Ω-loop (Figure 6C). Importantly, deep learning demonstrated that this pocket corresponds to a recurrent metastable ensemble rather than a rare fluctuation, providing a concrete allosteric hypothesis. Stabilisation of this constricted state could lock PDC-3 in a catalytically impaired conformation, offering a strategy for inhibitor design that circumvents the conserved orthosteric site.

## Class D β-lactamases

Class D β-lactamases (DBLs), referred to as OXA enzymes, are a group of SBLs that have gained considerable attention in recent years due to their increasing role in carbapenem resistance among Gram-negative pathogens. While most DBLs share a conserved overall tertiary structure (a characteristic α/β/α fold), they exhibit the greatest genetic heterogeneity among the four β-lactamase molecular classes. In fact, highly divergent members of this family frequently share less than 20% amino acid sequence identity. To address the inconsistencies in residue numbering caused by this extreme diversity, a standardised structural alignment-based numbering scheme was recently established [11]. This unified framework includes an automated workflow to facilitate its adoption across the research community, streamlining cross-study comparisons and structural analyses. Implementing such standardised framework to refer to conserved residues across subfamilies is essential to guarantee consistency across future literature, ensuring that the data are primed for natural language processing models to effectively mine scientific records and accelerate drug discovery. DBLs exhibit several defining structural elements that distinguish them from other SBLs [11]. The catalytic serine forms an acyl-enzyme intermediate with β-lactam substrates, and deacylation is facilitated by a conserved carboxylated lysine residue that replaces the canonical Glu or Asp general base found in classes A and C [5,90]. The carboxylation state of this Lys is essential to catalytic competence, and subtle perturbations of its microenvironment can modulate activity [5,91]. In addition, substrate recognition is shaped by a set of flexible loops, including the B5–B6 loop and Ω-loop analogues [11]. These local structural elements contribute to the remarkable functional diversity observed within Class D enzymes.

**Figure 7:**
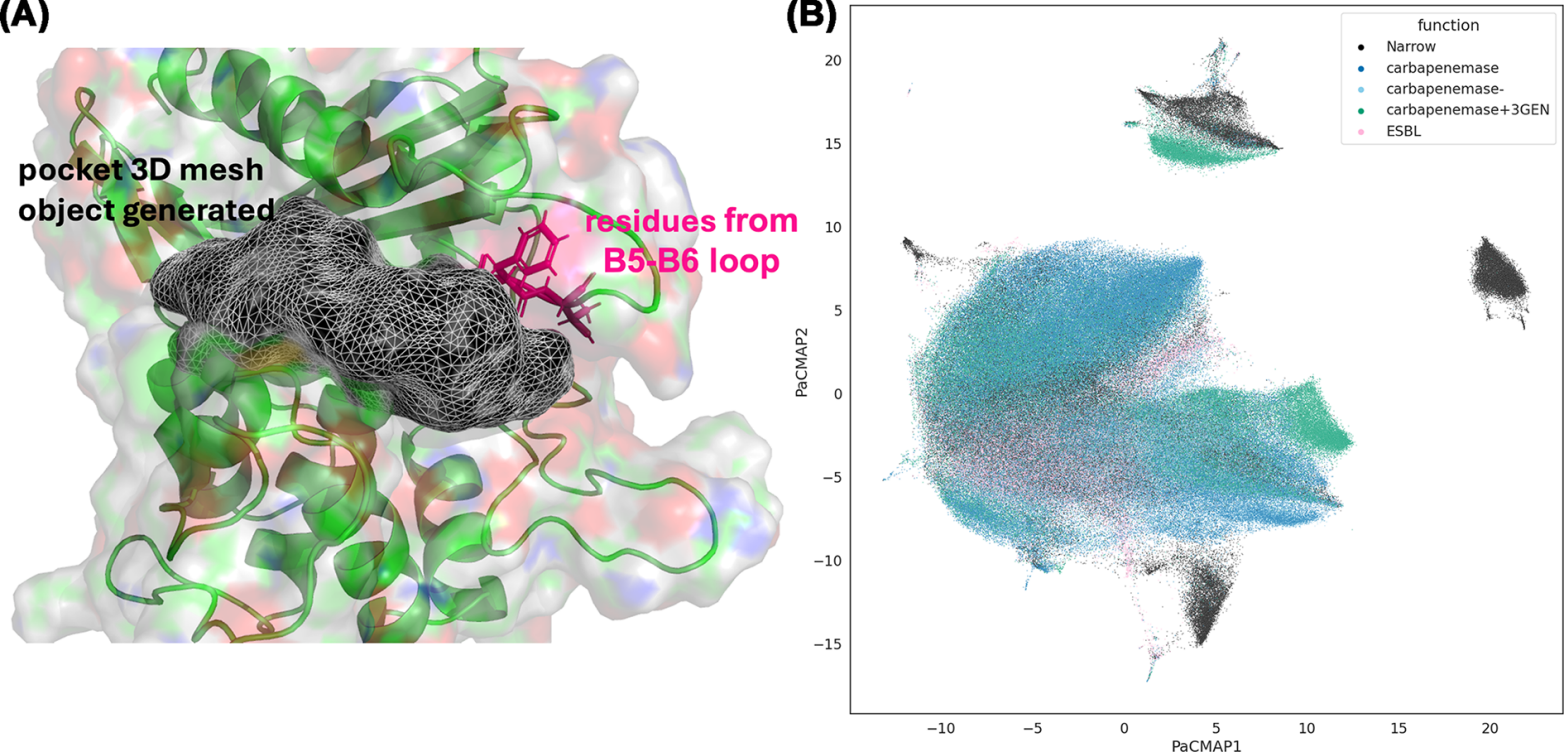
Structural illustration of active-site pocket extraction of Class D β-lactamase (**A**) A three-dimensional mesh representing the computed substrate-binding pocket is shown in black within the enzyme structure (green cartoon and semi-transparent surface). Residues belonging to the B5–B6 loop, which contribute to pocket shape and substrate accommodation, are highlighted in magenta. (**B**) Functional annotation mapped onto the PaCMAP space. Conformations are coloured by experimentally annotated function. Functional classes partially segregate within embedding space, illustrating relationships between sequence variation and enzymatic activity.

Recent computational and structural studies, including binding-site-centred analyses, have revealed that some functional heterogeneity of OXA enzymes arises from local differences in pocket geometry and physicochemical composition [11]. The active-site architecture is defined by a conserved core cluster of residues surrounding the catalytic serine; however, adjacent loops, particularly the B5–B6 loop, introduce structural variability (Figure 7A). Inclusion or exclusion of these residues in computational pocket definitions has been shown to strongly influence clustering and similarity mapping, highlighting their importance in shaping substrate access, recognition, and stabilisation of intermediates.

Consistent with these observations, analyses of 3D pocket embeddings demonstrate that OXA enzymes from the same subfamily tend to cluster together, reflecting shared local features. However, clear local structural distinctions are observed across subfamilies, supporting the idea that the OXA family comprises multiple evolutionary trajectories rather than a single structurally homogeneous group. Notably, even functionally similar carbapenemases can exhibit distinct pocket geometries, suggesting convergent evolution toward carbapenem hydrolysis mediated by different structural solutions.

Despite broad phylogenetic divisions, Class D enzymes display substantial functional overlap. ESBL-type OXAs form partially overlapping clusters with carbapenemases when viewed through structure-based embeddings, implying that factors beyond immediate binding-site geometry, such as global dynamics, active-site solvation, or oligomeric state, also contribute to substrate selectivity (Figure 7B). Conversely, narrow-spectrum OXAs are more structurally distinct, forming isolated clusters consistent with their limited substrate range. These trends emphasise that functional evolution within Class D β-lactamases arises from a combination of local pocket remodelling and broader conformational adaptability.

The structural plasticity of Class D β-lactamases poses challenges for predicting resistance phenotypes from sequence alone. Approaches centred on local binding-site features, such as 3D pocket embeddings derived from deep learning models, have shown the ability to capture subtle structural differences across large enzyme sets in other classes. Their ability to resolve subfamily-level similarities while highlighting functional divergence highlights their potential utility for surveillance, variant annotation, and mechanistic hypothesis generation.

## Machine learning approaches to probe catalytic mechanisms and functional state differentiation

Although deep learning has recently gained attention in β-lactamase research, classical machine-learning (ML) methods have also been applied to elucidate β-lactamase structure-function relationships [92], catalytic mechanisms [93], and functional state differentiation [94,95]. Four representative investigations illustrate the diversity and impact of these non-deep learning ML approaches.

A supervised projection-pursuit ML framework was implemented to investigate functional mechanisms [96,97], including those underlying antibiotic resistance in TEM-52 β-lactamase. By pairing experimentally categorised molecular systems with molecular-dynamics ‘digital twin’ simulations, the study uses feature extraction and supervised pattern recognition to identify mechanistic signatures governing enzyme function. This ML-guided workflow decomposes complex molecular behaviours into interpretable basis vectors and iteratively refines a data-driven working hypothesis. Applied to TEM-52, the approach successfully identifies mechanistic determinants of resistance, demonstrating the utility of ML in extracting meaningful structure-function insights from large biochemical datasets.

The QM/MM minimum-energy pathway simulations were integrated with interpretable ML to clarify the structural features controlling carbapenem deacylation in KPC-2 β-lactamase [93]. KPC-2 is one of the most prevalent class A carbapenemases worldwide and represents a major threat to the clinical efficacy of carbapenem antibiotics. Structural and simulation work shows that KPC-2’s active site is not rigid: subtle rearrangements of binding site residues, and the surrounding hydrophobic networks modulate the size and shape of the active site, enabling accommodation and efficient hydrolysis of diverse β-lactam substrates [74]. A tree-based ensemble model was trained to predict activation barriers for imipenem deacylation using active-site conformational descriptors. SHAP-based interpretability revealed that hydrogen-bonding interactions involving the general base and tautomerisation of the carbapenem pyrroline ring collectively modulate the reaction barrier [98]. This approach demonstrated how ML can extract interpretable mechanistic insights from high-dimensional QM/MM datasets, thereby accelerating mechanistic discovery in β-lactamase catalysis.

Complementing the mechanistic focus, supervised ML classification was used to differentiate functional binding modes of TEM-1 β-lactamase across different catalytic states [94]. Conventional dimensionality-reduction approaches such as PCA fail to reliably distinguish binding states [25]. However, supervised ML models successfully differentiated reactant versus product, apo versus product, and, to a lesser extent, apo versus reactant states. Feature-importance analysis indicated that residues Ser70 and Ser130 play pivotal roles in distinguishing key catalytic states, whereas other active-site residues contribute more substantially to apo/product discrimination.

Unsupervised ML methods are also particularly effective for clustering tasks because they do not require labelled data. PathDetect-SOM is a modified implementation of an unsupervised ML approach based on the Self-Organising Map (SOM), which clusters and visualises high-dimensional data by projecting it onto a two-dimensional grid while preserving similarities between data points [99]. During training, neighbouring neurones on the grid learn to represent similar input patterns, resulting in similar data points being positioned close together on the map. PathDetect-SOM has been successfully applied to investigate the binding kinetics of LP06, a potent inhibitor of PDC-3 β-lactamase [100]. Through pathway clustering, hydrophobic interactions were identified as critical for inhibitor recognition, particularly involving residues in the P2 loop (L119 and Q120), the Ω-loop (V211 and Y221), and the R2 loop (A292 and L293).

## Conclusions

Deep learning has fundamentally transformed our understanding of β-lactamase conformational dynamics, shifting the analytical focus from static structural comparison to the dynamic exploration of high-dimensional conformational landscapes. Across classes A–D β-lactamases, these approaches consistently show that resistance frequently emerges from subtle reshaping of free-energy surfaces rather than large-scale structural rearrangements.

DiffNets highlight causative structural determinants by coupling reconstruction with functional labels and EM-refined frame-level inference, revealing sub-angstrom features linked directly to phenotype. In parallel, CVAEs provide a robust, unsupervised means to reconstruct nonlinear conformational manifolds, identify metastable states, and expose cryptic conformations relevant to catalysis and inhibition. BindSiteS-CNN complements these global methods by offering a rotation-equivariant, binding site-focused view that resolves local structural changes driving specificity. CVAE-derived global embeddings and pocket-focused models provide a multi-scale view of how long-range dynamic coupling across the enzyme scaffold shapes functional divergence.

Importantly, the practical utility of these methods has been demonstrated through quantitative assessments in the cited studies. Supervised models such as DiffNets are evaluated using standard classification metrics and show clear advantages over unsupervised linear techniques such as PCA or tICA in separating functional states. CVAE-based analysis is validated through reconstruction accuracy, recovery of known metastable basins and direct comparisons with experimentally resolved conformations or catalytic intermediates. Binding-site-focussed CNNs further confirm their predictions through benchmarking against ligand-bound crystal structures and experimentally characterised specificity profiles. These performance evaluations support the conclusion that deep learning methods move beyond qualitative description and offer measurable improvement in capturing functionally relevant dynamics.

Despite these advances, several limitations remain. All approaches are intrinsically dependent on the quality, diversity, and convergence of the underlying MD sampling; inadequate exploration of conformational space can bias learned representations and obscure rare but functionally critical states. Model transferability across divergent β-lactamase classes is also a challenge, as architectures trained on one evolutionary background may not generalise without retraining or domain adaptation. In addition, model sensitivity to input representations, such as distance matrices versus coordinate-based or graph-derived features, can influence both interpretability and performance, emphasising the need for systematic benchmarking across featurisation strategies.

While the current application of DiffNets is limited by its reliance on binary functional labels, this framework is naturally extensible. Multi-class classifications, continuous-label regression (e.g., catalytic efficiency or resistance gradients), or hybrid supervised-unsupervised variants offer promising routes to capture more nuanced structure-function relationships without sacrificing interpretability. Such extensions would further enhance the ability of DiffNets to resolve functional continua rather than discrete states.

Together with enhanced sampling MD, deep learning establishes a coherent, data-driven method that moves beyond descriptive structural biology toward predictive modelling of conformational adaptation. As force-field accuracy improves, experimental validation strategies expand, and neural architectures continue to mature, deep learning-guided exploration of enzyme dynamics will become increasingly central to rational inhibitor design. By revealing exploitable metastable states and dynamic vulnerabilities, these approaches offer a powerful approach for the development of next-generation therapeutics aimed at combating AMR.

## Open Access

Open access for this article was enabled by the participation of University College London in an all-inclusive Read & Publish agreement with Portland Press and the Biochemical Society under an agreement with JISC.

## Abbreviations

AMR: antimicrobial resistance
DCI_asym_: asymmetric dynamic coupling index
CVAEs: convolutional variational autoencoders
ENM: elastic network model
EM: expectation–maximisation
GNN: graph neural networks
KL: Kullback–Leibler
ML: machine-learning
MBLs: metallo-β-lactamases
MD: molecular dynamics
PaCMAP: Pairwise Controlled Manifold Approximation
PCA: principal component analysis
SOM: Self-Organising Map
SBLs: serine β-lactamases
tICA: time-lagged independent component analysis
